# Tetraploid embryo aggregation produces high-quality blastocysts with an increased trophectoderm in pigs

**DOI:** 10.3389/fcell.2023.1239448

**Published:** 2023-11-16

**Authors:** Joohyeong Lee, Lian Cai, Mirae Kim, Hyerin Choi, Dongjin Oh, Ali Jawad, Eunsong Lee, Sang-Hwan Hyun

**Affiliations:** ^1^ Department of Companion Animal Industry, College of Healthcare and Biotechnology, Semyung University, Jecheon, Republic of Korea; ^2^ Laboratory of Veterinary Embryology and Biotechnology (VETEMBIO), Veterinary Medical Center and College of Veterinary Medicine, Chungbuk National University, Cheongju, Republic of Korea; ^3^ Institute of Stem Cell and Regenerative Medicine (ISCRM), Chungbuk National University, Cheongju, Republic of Korea; ^4^ Graduate School of Veterinary Biosecurity and Protection, Chungbuk National University, Cheongju, Republic of Korea; ^5^ College of Veterinary Medicine, Kangwon National University, Chuncheon, Republic of Korea

**Keywords:** tetraploid, blastomere aggregation, trophectoderm, parthenogenetic activation, pig

## Abstract

Tetraploid complementation is an ideal method for demonstrating the differentiation potential of pluripotent stem cells. In this study, we selected the most efficient tetraploid production method for porcine embryos and investigated whether tetraploid blastomere aggregation could enhance the quality of tetraploid embryos. Three methods were investigated to produce tetraploid embryos: First, tetraploid embryos were produced using electro-fusion of two-cell stage parthenogenetic blastomere (FUTP). Second, somatic cell was injected into the mature oocyte and fused to produce tetraploid embryos. Third, oocytes were matured with Cytochalasin B (CB) for the late 22 h of *in vitro* maturation to inhibit the first polar body (PB1). Following that, non-PB1 oocytes were treated with CB for 4 h after parthenogenetic activation. There was no significant difference in the blastocyst development rate and tetraploid production rate of the embryos produced through the three methods. However, FUTP-derived blastocysts had a significantly lower percentage of apoptotic cells compared to other methods. The developmental competence of embryos, expression of trophectoderm cell marker genes, and distribution of YAP1 protein were investigated in tetraploid embryos produced using the FUTP method. The FUTP method most effectively prevented apoptosis during porcine tetraploid embryo formation. Tetraploid aggregation-derived blastocysts have a high proportion of trophectoderm with increased expression of the CDX2 mRNA and high YAP1 intensity. High-quality blastocysts derived from a tetraploid embryo aggregation can serve as suitable source material for testing the differentiation potential of pluripotent stem cells for blastocyst complementation in pigs.

## 1 Introduction

Blastocyst complementation refers to the injection of cells into a blastocoel. This technique allows the production of chimeric animals (Wu et al., 2016), which have the potential to be used as an unlimited source of organs (Oldani et al., 2017). Tetraploid complementation is a method that ideally proves the differentiation potential of pluripotent stem cells such as embryonic stem (ES) or induced pluripotent stem (iPS) cells. Pluripotent stem cells can differentiate into cells of all tissues except placental tissue. When pluripotent stem cells are combined with embryos capable of differentiating into placental tissue, they can be grown into whole animals. Tetraploid complementation allows pluripotent stem cells to be produced as a complete organism by supplementing embryos capable of forming placental tissue. Previous studies have shown that the mouse naïve pluripotent stem cells can pass the tetraploid complementation test (Zhou et al., 2015; Li et al., 2017). However, mammalian species other than rats and mice have not yet succeeded in producing offspring using the tetraploid complementation method, and pluripotency of the injected cells has not been demonstrated.

Animal models for human disease are essential in medical research. Pigs are more anatomically and physiologically similar to humans than small rodents such as mice; therefore, pigs are an attractive option for modeling human disease (Walters and Prather, 2013). Recent advances in genetic engineering have facilitated the rapid expansion of pig models for the study of human disease (Hou et al., 2022). However, the production of ES cells, representative pluripotent stem cells, has not been established in pigs (Brevini et al., 2010; Vassiliev et al., 2010; Hou et al., 2016). The published cell lines usually do not meet the stringent criteria for pluripotency and are frequently called “ES-like” cells. Besides studies on the production of pluripotent cell lines in pigs, extensive investigations are required to enhance the quality of blastocyst used for tetraploid complementation.

In general, embryos produced *in vitro* are inferior to *in vivo*-derived embryos (Holm et al., 2002; Stokes et al., 2005). Pig embryos used in assisted reproductive technology are mostly derived from *in vitro*-derived oocytes. The low-quality *in vitro*-derived embryos often leads to the low-quality donor blastocysts used for tetraploid complementation. In addition, the total number of cells in a tetraploid blastocyst is lower than that in a diploid blastocyst (Ishiguro et al., 2005; Kawaguchi et al., 2009; Park et al., 2011). Therefore, a method to overcome these disadvantages must be investigated to produce good-quality embryos.

Several researchers have studied various methods of tetraploid embryo production. The most commonly used method in mammalian species is the production of blastomere of a two-cell stage embryo by fusing (Curnow et al., 2000; Krivokharchenko et al., 2002; Atikuzzaman et al., 2011; Kong and Liu, 2019). A method for producing tetraploid embryos by inserting a somatic cell nucleus into an intact metaphase II (MII) oocyte followed by activation and Cytochalasin B (CB) treatment has been introduced (Fu et al., 2016). Another method for producing tetraploid embryos is to inhibit the extrusion of the first polar body (PB1) and second polar body (PB2) through CB treatment during maturation and after activation (Lin et al., 2019). It is necessary to determine which of these methods for producing tetraploid embryos in pigs is the most effective.

The embryo aggregation method is widely used in various mammals to improve production efficiency by compensating for developmental deficits of *in vitro*-derived embryos (Lee et al., 2022). This strategy consists of placing two or more zona pellucida (ZP)-free embryos in close proximity during *in vitro* culture so that the blastomeres self-organize to form one single blastocyst of improved quality (Savy et al., 2021). These improvements are due to “epigenetic compensation” between higher cell numbers, aggregate structures, or both early in development (Boiani et al., 2003; Eckardt and McLaughlin, 2004). The blastomere aggregation method can be an effective tool to improve low-quality tetraploid embryos. However, whether aggregated tetraploid porcine blastocysts have characteristics such as morphology, blastocyst cell composition, and expression of transcription factors remains unknown.

In this study, we selected the most efficient tetraploid production method for porcine embryos and investigated whether blastomere aggregation could improve the quality of tetraploid embryos. Our findings help gain insight into the evolution and regulation of pluripotency across mammalian species.

## 2 Materials and methods

### 2.1 Ethics statement and animal information

The animal experiments were reviewed, and the experimental protocol was approved by the Committee on Ethics of Animal Experiments of the Chungbuk National University (Permit Number: CBNUA-1733-22-01). The animals employed in this study were treated in compliance with the standard operating procedures set forth by the Institutional Animal Care and Use Committee. The specific anesthesia and analgesia protocols are delineated in the following subsections. The authors of this article further confirm that the experiments, statistical analyses, and information presented here adhere to the recommendations outlined in the guidelines of Animal Research: Reporting of *in vivo* Experiments.

### 2.2 Culture media

All chemicals were purchased from Sigma-Aldrich Corporation (St. Louis, MO, United States) unless otherwise stated. Oocytes were matured in Medium-199 (Invitrogen, Grand Island, NY, United States) supplemented with 0.91 mM pyruvate, 0.6 mM cysteine, 10 ng/mL epidermal growth factor, and 1 μg/mL insulin, and 10% (v/v) porcine follicular fluid was used as basal medium for *in vitro* maturation (IVM). Porcine zygote medium (PZM)-3 containing 0.3% (w/v) bovine serum albumin (BSA) was used as *in vitro* culture (IVC) medium. On day 4, we transferred embryos into PZM-3 droplets containing 10% fetal bovine serum (Thermo Fisher Scientific, Waltham, MA, United States).

### 2.3 Experimental design

**FIGURE 1:**
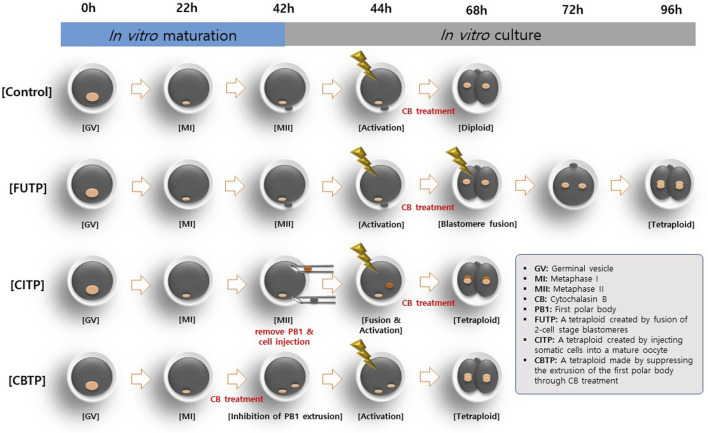
Different methods for generating tetraploid embryos in pigs. Tetraploid embryos were produced by electro-fusion of two-cell stage PA embryos (FUTP). The diploid somatic cell was injected into the mature oocyte and fused to produce tetraploid embryos (CITP). Oocytes were matured with CB for the late 22 h of *in vitro* maturation to inhibit the PB1. Thereafter, non-PB1 oocytes were treated with CB for 4 h after parthenogenetic activation (CBTP).

In the first experiment, we evaluated the preimplantation developmental competence of embryos using three methods to produce tetraploid as shown in Figure 1: First, tetraploid embryos were produced using electro-fusion of the two-cell stage parthenogenetic activation (PA) blastomere (FUTP). Second, the diploid somatic cell (porcine ear fibroblasts) was injected into the mature oocyte and fused to produce tetraploid blastomere (CITP). Third, oocytes were matured with 5 μg/mL CB for the late 22 h of IVM to inhibit the PB1. Subsequently, non-PB1 oocytes were treated with CB for 4 h after PA (CBTP). In the second experiment, the proportion of blastocysts constructed as tetraploids were investigated by analyzing the nuclear ploidy of the blastocysts aggregated through the three tetraploid production methods. In the third experiment, the apoptotic cell rates of blastocysts were investigated according to the three methods of producing tetraploid embryos. In the fourth experiment, the diameter and surface area of blastocysts produced through embryo aggregation was calculated. In the fifth experiment, the developmental competence of embryos was investigated through the aggregation of tetraploid embryos produced by the FUTP method. In the sixth experiment, the expression of pluripotency and trophectoderm (TE) cell markers genes in aggregated embryo-derived blastocysts were investigated. In the seventh experiment, the distribution of YAP1 protein in aggregated embryo-derived blastocysts was investigated.

### 2.4 Oocyte collection and IVM

The IVM of oocytes was performed in the same manner as described by Lee et al. (Lee et al., 2021). The ovaries of prepubertal gilts obtained from a local abattoir were transported to the laboratory at 34°C–37°C. Cumulus oocyte complexes (COCs) were aspirated from follicles with diameters ranging from 3 to 8 mm. COCs with multiple layers of compacted cumulus cells were selected and washed three times in HEPES-buffered Tyrode’s medium (TLH) containing 0.05% (w/v) polyvinyl alcohol (TLH-PVA). COCs were placed in each well of a four-well multi-dish (Nunc, Roskilde, Denmark). Each well contained 500 µL of IVM with 10 IU/mL equine chronic gonadotropin and 10 IU/mL human chorionic gonadotropin (Intervet, Boxmeer, Netherland). After 22 h, the COCs were transferred to a fresh IVM medium, without equine or human chorionic gonadotropin, and incubated for 22 h. IVM was performed at 39°C and 5% CO_2_ in a humid incubator (Astec, Fukuoka, Japan). For the CBTP treatment group, oocytes were cultured in an IVM medium supplemented with 5 μg/mL CB for the last 22 h of IVM. After IVM, COCs were denuded by gentle pipetting with 0.1% hyaluronidase.

### 2.5 Assessment of nuclear status of oocyte and chromosome ploidy of blastocyst

The nuclear status of oocytes was assessed in the same manner as described by Cai et al. (2022). The nuclear state of oocytes derived from the CBTP-treated group was examined. The denuded oocytes were washed thrice in TLH-PVA medium and stained with 5 μg/mL Hoechst 33,342 in TLH-PVA for 10 min. The stained oocytes were examined using an epifluorescence microscope (Nikon, Tokyo, Japan) with an ultraviolet (UV) filter (370 nm) and were classified according to the meiotic maturation stage as germinal vesicles (GV), or being in the metaphase I (MI), anaphase I/telophase I (AI/TI), or MII stage. The blastocysts derived from each treatment group were subjected to karyotyping to confirm the chromosome ploidy. Porcine blastocysts were treated with 0.4 μg/mL demecolcine for 6 h. Blastocysts were then added to a hypotonic solution (0.075 M KCl). Swollen blastocysts were fixed on a clean glass slide immersed in a fixative (acetic acid/methanol = 1:3 v/v). Chromosome spreads were stained with 10% (v/v) Giemsa before imaging.

### 2.6 Donor cell injection and fusion

Before the experiments, one male Jeju mini pig weighing 30–45 kg was provided *ad libitum* access to food and water and fasted for 12 h. For skin biopsies, 10 mg/kg ketamine hydrochloride (Yuhan, Seoul, Korea) was intramuscularly injected, followed by initial sedation, and respiratory anesthesia was maintained by using Isoflurane (Hana Pharm, Seoul, Korea). Skin ear tissue was recovered from three places using a 3-mm biopsy punch (Kai industries, Tokyo, Japan) and sterilized with 10% povidone iodine and 70% alcohol. Sufficient hemostasis and antibiotic ointment were applied post biopsy to prevent infection. For the CITP treatment group, porcine ear fibroblasts or cumulus cells were inserted into the MII oocyte. Porcine ear fibroblasts were cultured in Dulbecco’s modified Eagle’s medium F-12 (Invitrogen) supplemented with 10% (v/v) fetal bovine serum until a complete monolayer of cells was formed. A suspension of single cells was prepared by trypsinization using EDTA-trypsin and resuspending in TLH containing 0.4% (w/v) bovine serum albumin (TLH-BSA) before cell injection. IVM oocytes were incubated for 10 min in TLH-BSA medium, washed twice with fresh TLH-BSA medium, and then transferred to a droplet of TLH-BSA containing 5 μg/mL CB covered with mineral oil. The PB1 were enucleated aspiration using a 16-μm polar body biopsy pipette (sunlight Medical, FL, United States). A donor cell was inserted into the perivitelline space of an MII oocyte. The reconstituted single cell-oocyte couplets were simultaneously induced to fuse and activate through a single electrical stimulation. Fusion-activation was induced by applying an alternating current field of 2 V at 1 MHz for 2 s, followed by two pulses of 170 V/mm direct current for 30 μs using an Electro Cell Fusion Generator (LF101; NepaGene, Chiba, Japan) in a fusion-activation medium containing 280 mM mannitol, 0.05 mM MgCl_2_, and 0.1 mM CaCl_2_. After electrical stimulation, oocytes were transferred to TLH-BSA medium and fused oocyte was checked 30 min later. Fused oocytes were transferred to PZM-3 supplemented with 5 μg/mL CB for 4 h at 39°C temperature and in a humidified atmosphere of 5% CO_2_, 5% O_2_, and 90% N_2_. Embryos were transferred to PZM-3 and cultured until the next step at 39°C temperature in a humidified atmosphere of 5% CO_2_, 5% O_2_, and 90% N_2_.

### 2.7 PA and blastomere fusion

The PA of oocytes for FUTP and CBTP methods were performed as previously described by Lee et al. (Lee et al., 2021). Only oocytes from which the PB1 was released after IVM were selected for PA. They were placed between two wires in a 1-mm fusion chamber coated with a fusion-activation medium. Oocyte activation was stimulated with a direct current pulse of 120 V/mm for 60 μs using a Cell Fusion Generator. Electrically stimulated oocytes were transferred to PZM-3 with 5 μg/mL CB and cultured for 4 h at 39°C temperature and in a humidified atmosphere of 5% CO_2_, 5% O_2_, and 90% N_2_. PA embryos were transferred to PZM-3 and cultured until the next step at 39 °C temperature in a humidified atmosphere of 5% CO_2_, 5% O_2_, and 90% N_2_. To produce tetraploid embryos through the FUTP method, only embryos with evenly divided blastomeres were selected 24 h after *in vitro* culture. Two-cell stage embryos were transferred to an electro-fusion chamber coated with fusion-activation medium. The division plane of the embryo was placed horizontal to the electrode and then two pulses of 170 V/mm direct current for 30 μs were supplied using an electric cell fusion generator. After electrical stimulation, 2-cell stage embryos were transferred to PZM-3 and fusion of blastomeres was checked 30 min later.

### 2.8 ZP removal and blastomere aggregation

According to the experimental design, the two-cell stage PA embryos were collected 24 h after electrical activation. The collected embryos were incubated with acidic Tyrode′s Solution (TS) to remove the ZP, as previously described by Lee et al. (Lee et al., 2022). The collected embryos were incubated in 50% TS solution for 1 min, then in 100% TS solution for 30 s, and then sufficiently removed using a glass pipette. The ZP-free blastomeres were aggregated using phytohemagglutinin-L (PHA-L) (Lee et al., 2022). The blastomeres were aggregated by culturing for 20 min in an IVC medium with 15 μg/mL PHA-L. Aggregated blastomeres were transferred to PZM-3 and cultured for 144 h at 39°C in a humidified atmosphere of 5% CO_2_, 5% O_2_, and 90% N_2_.

### 2.9 Evaluation of developmental competence of blastocyst and total cell count

Day 0 was regarded as the day on which PA was initiated. On day 1, cleavage formation was analyzed. Blastocyst formation was assessed on day 7. To calculate the total cell number of blastocysts on day 7, the blastocysts were washed in TLH-PVA and fixed in 4% (v/v) paraformaldehyde in phosphate-buffered saline containing 0.05% PVA and stained for 10 min with 5 μg/mL of Hoechst-33342. Then, the blastocysts from each group were transferred to a drop of 100% glycerol on a glass slide and gently covered with a coverslip. The stained blastocysts were observed using a fluorescence microscope (Nikon, Tokyo, Japan) at ×200 magnification.

### 2.10 Terminal deoxynucleotidyl transferase dUTP nick-end labeling (TUNEL) assay

Analysis of apoptosis was performed as previously described by Lee et al. (Lee et al., 2022). To analyze apoptosis in blastocysts, they were fixed with 4% (v/v) paraformaldehyde for 1 h at room temperature, washed with Dulbecco’s phosphate-buffered saline (DPBS) containing 0.05% PVA, and permeabilized with 0.1% (v/v) Triton X-100 in 0.1% (w/v) sodium citrate for 1 h at 20°C–25°C. After rinsing with DPBS containing 0.05% PVA, the embryos were stained with 45 μL of TUNEL-Label solution (Roche, Mannheim, Germany) supplemented with 5 μL TUNEL-Enzyme solution (Roche) for 1 h at 39°C in a dark, humidified atmosphere. Subsequently, the nuclei were stained with 5 μg/mL Hoechst-33342 for 10 min.

### 2.11 Diameter measurement and surface area conversion of blastocyst

Blastocysts in each group were recorded at ×2 magnification using a digital camera (DS-L3; Nikon) attached to an inverted microscope (TE-300; Nikon). The diameter of the blastocyst was measured using the ImageJ software, version 1.46 (National Institutes of Health, Bethesda, MD, United States). The surface area of the blastocyst was converted based on the diameter of the blastocyst described in Supplementary Figure S1.

### 2.12 Gene expression analysis via quantitative real-time polymerase chain reaction (qRT-PCR)

qRT-PCR was performed as previously described by Lee et al. (Lee et al., 2022). The relative expression of porcine pluripotency-related genes (*POU5F1*, *NANOG*, and *SOX2*) and TE markers (*CDX2*) in aggregated tetraploid blastocysts was investigated. The blastocysts were washed once in DPBS, centrifuged, and the supernatant was removed and stored at −80°C until analysis. Gene expression was analyzed using the CFX96 Touch Deep Well Real-Time PCR Detection System (BIO-RAD, Hercules, CA, United States). After mRNA extraction and cDNA synthesis, qRT-PCR was performed using 2 μL of cDNA template with 10 μL of 2X SYBR Premix Ex Taq (Takara Bio Inc., Shiga, Japan) containing primers specific to *CDX2, POU5F1, SOX2*, and *NANOG* (Supplementary Table S1). Reactions were performed for 40 cycles under the following conditions: denaturation at 95°C for 30 s, annealing at 57°C for 15 s, and extension at 72°C for 30 s. Gene expression was quantified relative to the reference gene *RN18S*. Relative quantification was based on a comparison of the threshold cycle (Ct) at a constant fluorescence intensity. Relative mRNA expression was calculated using the following equation: R = 2^-[ΔCt sample−ΔCt control]^. Expression values were normalized to those of *RN18S*.

### 2.13 Immunofluorescence analysis

Immunofluorescence analysis was performed as previously described by Lee et al. [21]. For immunofluorescence analysis, blastocysts fixed with 4% paraformaldehyde were washed three times with phosphate-buffered saline (PBS) and permeabilized with 0.5% Triton X-100 for 30 min. Then, blastocysts were co-incubated with blocking solution (10% goat serum in PBS) and primary antibody overnight at 4°C. The primary antibodies used were anti-SOX2, anti-CDX2, and anti-YAP1, described in Supplementary Table S2. After washing three times with a washing medium (Tween 20, Triton X-100, and PBS), the blastocysts were incubated with the appropriate secondary antibodies. Secondary antibodies were applied, and the cells were incubated at 20°C–25°C for 1 h. Nuclei were stained with Hoechst-33342. The stained blastocysts were observed using a fluorescence microscope (Nikon, Tokyo, Japan) at ×200 magnification.

### 2.14 Statistical analysis

Statistical analysis was performed using Statistical Analysis System software (version 9.4; SAS Institute, Cary, NC, United States). In experiments 1 to 3, the standard diploid embryo generation method and three tetraploid generation methods were analyzed as independent variables. Proportions of oocytes reached MII, cleavage rate, blastocyst rate, nuclear states, and TUNEL-positive nuclei were analyzed as dependent variables. In experiments 4 to 7, the standard diploid embryo generation method, FUTP, and 3X FUTP methods were analyzed as independent variables. Proportions of cells in blastocyst, diameter of blastocyst, surface area of blastocyst, gene expression, and YAP1 intensity were analyzed as dependent variables. The data were subjected to analysis of variance (ANOVA) using a general linear model procedure. Post-hoc analyses to identify between-group differences were performed using the least significant difference test when treatments differed at a (*p* < 0.05). Percentage data were arcsine transformed prior to analysis to maintain homogeneity of variance. Results are expressed as mean ± standard error of the mean (SEM).

## 3 Results

### 3.1 Developmental competence of tetraploid embryos according to the methods of producing tetraploid

We investigated the *in vitro* developmental competence of embryos according to the tetraploid production method (Table 1). The average nuclear maturation rate of the oocytes used in control, FUTP, and CBPT was 83.5% ± 0.9%, and that of the CBTP group was 9.2% ± 2.1%. During the last 22 h of IVM, 96.7% ± 1.2% of CB-treated oocytes (CBTP) were arrested in the MI stage (Supplementary Table S3). In the CBTP group, there was no significant difference in the cleavage rate at 24 h after activation compared with that in the control group, but the ratio in the two-cell stage was significantly reduced (61.6% ± 4.0% vs. 51.4% ± 3.5%). The blastocyst development rate of the CITP group (55.9% ± 6.5%) was significantly (*p* < 0.05) lower than that of the control group (76.3% ± 4.7%), but there was no significant difference when compared to the FUTP and CBTP groups (62.7%–68.7%).

**TABLE 1:** *In vitro* developmental competence according to various methods for generating tetraploid embryos in pigs.

Treatment	No. (%) of oocytes that reached MII	No. (%) of oocytes fused	No. (%) of cleaved oocytes	No. (%) of two-cell	No. (%) of blastomere fused	No. (%) of embryos developed to blastocyst/two-cell
Control (Diploid)	910/1093 (83.5 ± 0.9)^a^	_	380/453 (75.3 ± 4.0)	283/453 (61.6 ± 4.0)^c^	_	96/122 (76.3 ± 4.7)^c^
FUTP		_			153/156 (98.1 ± 1.0)	100/153 (62.7 ± 4.5) ^cd^
CITP		276/407 (68.0 ± 6.0)	190/262 (71.3 ± 2.6)	141/262 (55.9 ± 6.5) ^cd^	_	84/141 (55.9 ± 6.5)^d^
CBTP	29/343 (9.2 ± 2.1)^b^	_	219/298 (72.3 ± 4.5)	157/298 (51.4 ± 3.5)^d^	_	109/157 (68.7 ± 5.4) ^cd^

Ten replicates.

^a,b^ Values in the same column with different superscript letters are significantly different (*p* < 0.01).

^c,d^ Values in the same column with different superscript letters are significantly different (*p* < 0.05).

### 3.2 Chromosome ploidy of the blastocysts according to the methods of producing tetraploid

**FIGURE 2:**
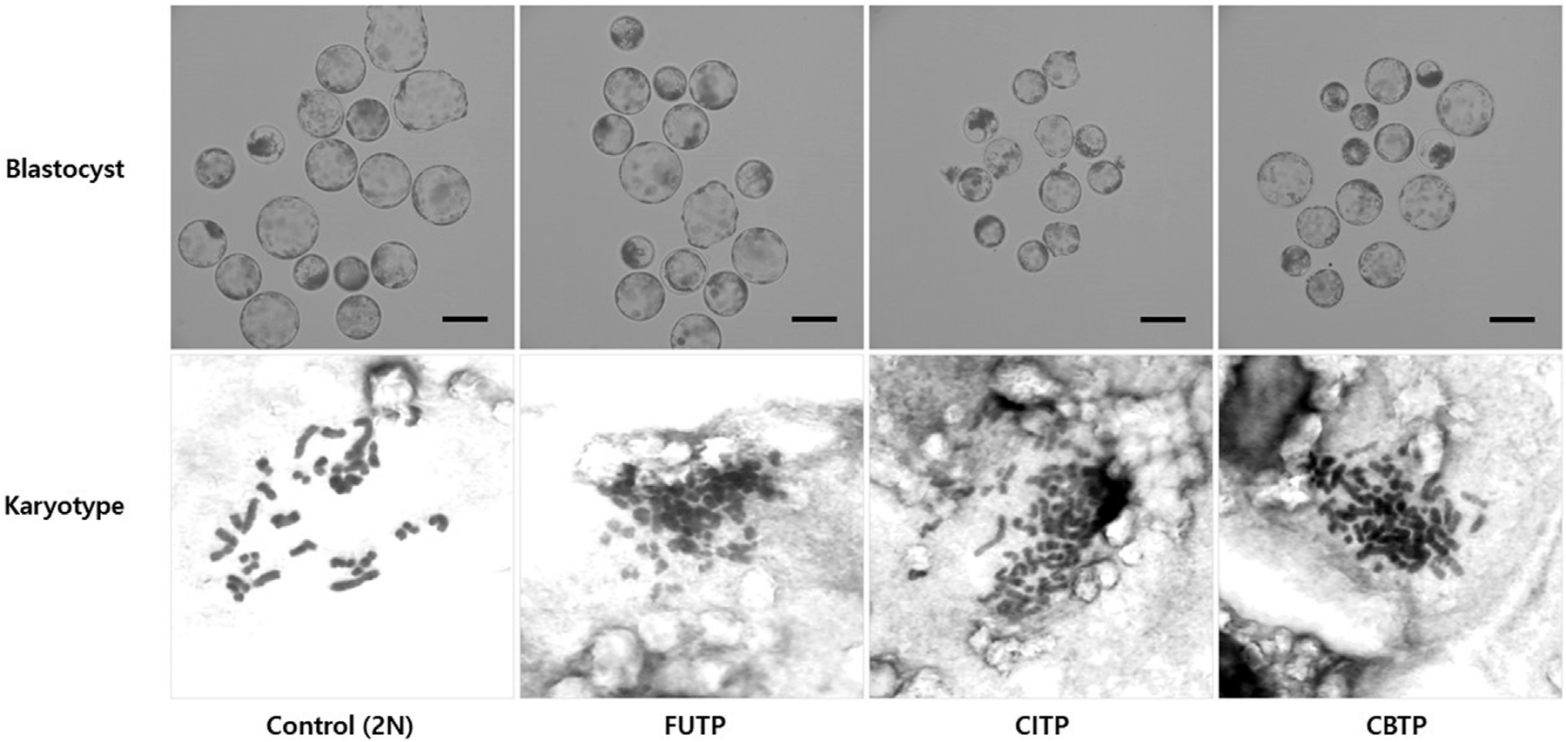
Morphology of blastocysts derived from various methods of producing tetraploid embryos. The chromosome ploidy of the blastocyst was observed at 400×, a blastocyst with about 38 chromosomes was recognized as a diploid, and a blastocyst with about 76 chromosomes as a tetraploid. Scale bar = 200 μm.

We analyzed the chromosome ploidy of blastocysts derived from the tetraploid production method (Table 2). The tetraploid production method groups showed a significantly (*p* < 0.01) lower diploid ratio (14.0%–25.4% vs. 84.6%) and a higher tetraploid ratio (74.6%–86.0% vs. 5.0%) than did the control group. Representative images of blastocysts and chromosome ploidy from each treatment group are shown in Figure 2.

**TABLE 2:** Nuclear state of blastocysts according to various methods for generating tetraploid embryos in pigs.

Treatment	No. of blastocysts evaluated *	Nuclear states (%)			
		Haploid	Diploid	Polyploid	Tetraploid
Control (Diploid)	20	2 (10.4 ± 6.3)^a^	17 (84.6 ± 5.4)^a^	0 (0.0 ± 0.0)	1 (5.0 ± 5.0)^a^
FUTP	21	0 (0.0 ± 0.0)^b^	3 (14.0 ± 5.2)^b^	0 (0.0 ± 0.0)	18 (86.0 ± 5.2)^b^
CITP	20	0 (0.0 ± 0.0)^b^	3 (17.1 ± 7.5)^b^	1 (2.5 ± 2.5)	16 (80.4 ± 7.1)^b^
CBTP	21	0 (0.0 ± 0.0)^b^	4 (25.4 ± 8.6)^b^	0 (0.0 ± 0.0)	17 (74.6 ± 8.6)^b^

*Four replicates.

^a,b^ Values in the same column with different superscript letters are significantly different (*p* < 0.01).

### 3.3 Apoptotic cell rates of blastocysts according to the methods of producing tetraploid

**FIGURE 3:**
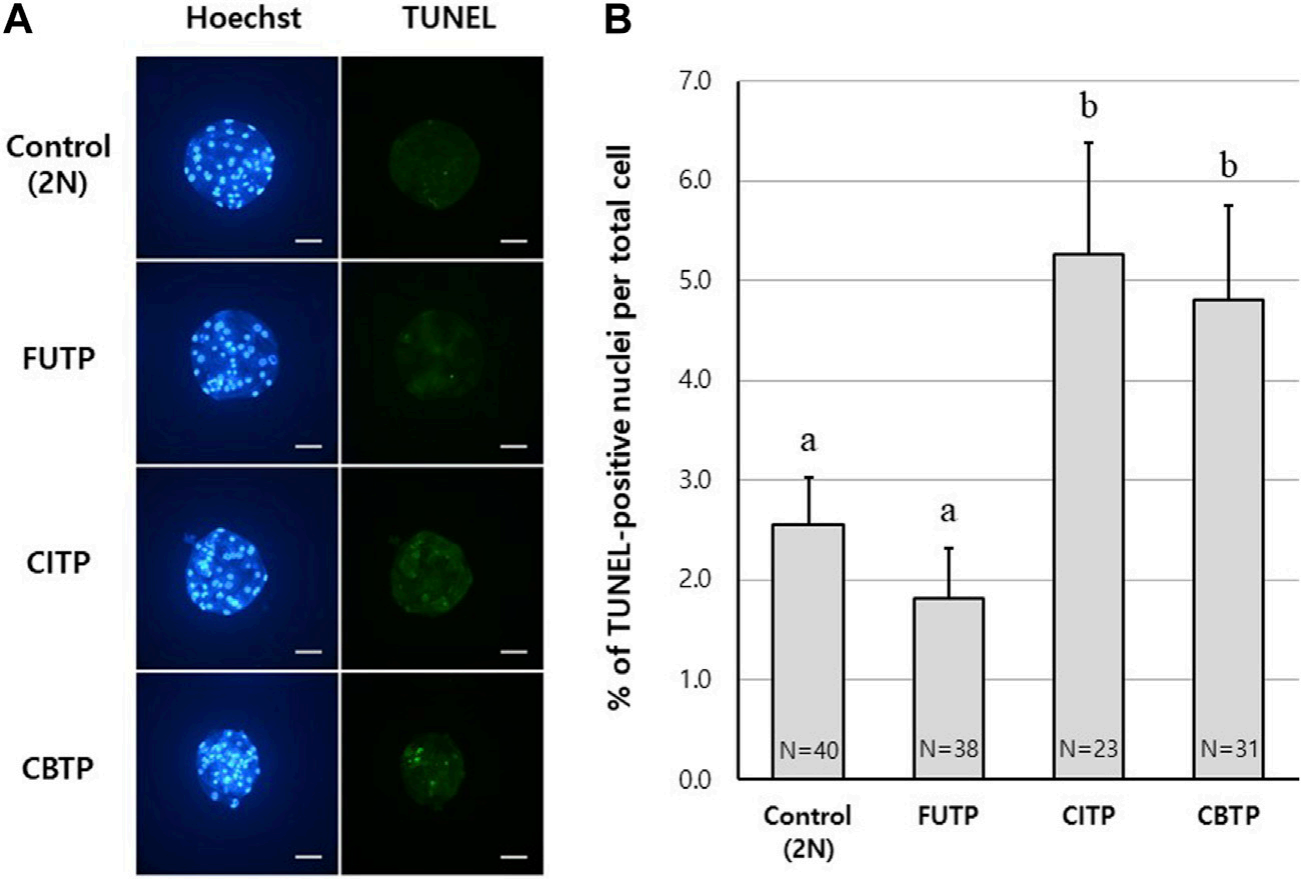
Apoptotic cell rates of blastocysts according to the methods of producing tetraploid. **(A)** Representative image of TUNEL assay according to tetraploid embryo-producing method. Scale bar = 50 μm. **(B)** Apoptotic cell index (%) according to the tetraploid embryo-producing method. N = the number of blastocysts analyzed. Data are the mean ± SEM. ^a, b^ Values in the same column with different superscript letters are significantly different (*p* < 0.05).

Control and tetraploid blastocysts were examined for the percentage of apoptotic cells using the TUNEL assay. Representative images of blastocyst nuclei and TUNEL-positive cells from each treatment group are shown in Figure 3A. The FUTP (1.8%) group showed significantly (*p* < 0.05) lower apoptotic cell rates than did the CITP (5.3%) and CBTP (4.8%) groups, but there was no significant difference from the control group (2.6%) (Figure 3B).

### 3.4 Diameter and surface area of blastocysts produced through embryo aggregation

We investigated the effect of tetraploid embryo aggregation on the size of the blastocyst, and the number of cells was compared with the surface area of the blastocyst (Tables 3, 4). The 3X FUTP group showed a significantly (*p* < 0.05) larger blastocyst diameter than did the control and FUTP groups (294.4 ± 9.8 μm vs. 190.9 ± 10.3 μm vs. 199.0 ± 11.3 μm). In addition, the nucleus in the tetraploid blastocysts showed a significantly (*p* < 0.05) larger diameter than that in the control group (19.2–19.4 μm vs. 14.1 μm). The average surface area of blastocysts was converted from the diameter of blastocysts derived from each treatment group. When the surface area of the blastocyst per cell in the control group was normalized to “1”, the FUTP and 3X FUTP groups were 1.43 and 1.34, respectively.

**TABLE 3:** Effect of tetraploid blastomere aggregation on blastocyst rate, cell number, the proportion of ICM and TE cells in blastocysts.

Type of blastomere	No. (%) of embryos [aggregate] developed to blastocyst/two-cell	No. of blastocyst evaluated *	No. of total cells in blastocyst	No. of ICM in blastocyst	ICM ratio (%)	No. of TE cell in blastocyst	TE cell ratio (%)
Control (Diploid)	55/77 (71.2 ± 3.9) ^ab^	30	39.2 ± 2.5^a^	6.4 ± 1.0^a^	14.7 ± 1.8^a^	32.8 ± 1.9^a^	85.3 ± 1.8^a^
FUTP	43/68 (63.1 ± 2.9)^a^	28	29.8 ± 2.1^b^	3.1 ± 0.4^b^	10.1 ± 1.6^b^	26.8 ± 1.9^a^	89.7 ± 1.6^b^
3X FUTP	42/[57] (77.2 ± 2.3)^b^	30	71.3 ± 3.9^c^	5.7 ± 0.7 ^ab^	8.2 ± 1.1^b^	65.5 ± 3.7^b^	92.5 ± 1.1^b^

*Six replicates.

^a,b,c^ Values in the same column with different superscript letters are significantly different (*p* < 0.05).

**TABLE 4:** Effect of tetraploid blastomere aggregation on diameter and surface area of the blastocyst.

Type of blastomere	No. of blastocysts evaluated *	Dimeter of blastocyst (µm)	No. of blastocyst nucleus evaluated	Diameter of the blastocyst nucleus (µm)	Surface area of blastocyst (µm^2^)	Surface area/cell (µm^2^)	Relative proportion^A^
Control (Diploid)	21	190.9 ± 10.3^a^	100	14.1 ± 0.3^a^	121,095 ± 13,406^a^	3,089	1
FUTP	21	199.0 ± 11.3^a^	100	19.2 ± 0.4^b^	132,356 ± 15,041^a^	4,442	1.43
3X FUTP	21	294.4 ± 9.8 ^b^	100	19.4 ± 0.4^b^	278,183 ± 18,643^b^	3,902	1.34

*Five replicates.

^a,b^ Values in the same column with different superscript letters are significantly different (*p* < 0.05).

^A^ The relative ratio of blastocyst surface area per cell was calculated by normalizing the control to “1”.

### 3.5 Developmental competence of aggregated tetraploid embryos

**FIGURE 4:**
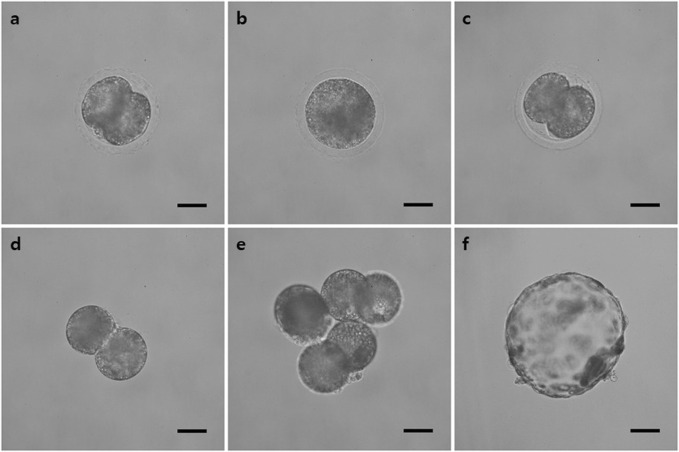
Representative image of the tetraploid blastocyst production process using the FUTP method. **(A)** Two-cell stage embryo 24 h after parthenogenetic activation; **(B)** embryo in which two blastomeres were fused through electrical stimulation; **(C)** re-cleaved embryos 24 h after fusion; **(D)** re-cleaved embryo with the zona pellucida removed; **(E)** Aggregated three re-cleaved embryos; **(F)** blastocysts of aggregated tetraploid embryos on day 7 after parthenogenetic activation.

**FIGURE 5:**
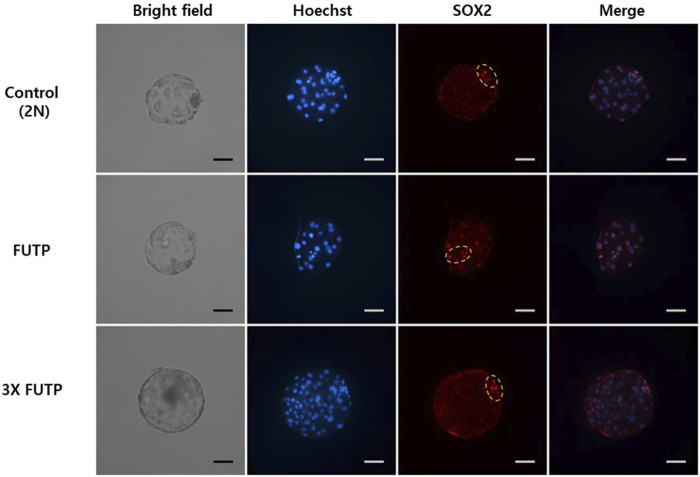
Representative images of fluorescent immunostaining. Nuclei (blue) and inner cell mass (red) of aggregated tetraploid blastocysts. The yellow dotted circle indicates the inner cell mass of the blastocyst.

The morphological changes in the blastocyst formation process through tetraploid embryo formation and blastomere aggregation are shown in Figure 4. The FUTP-derived tetraploid-derived embryos were cultured singly (FUTP) or in aggregates of three embryos (3X FUTP). The blastocysts derived from each group were examined for the number of inner cell mass (ICM) using SOX2 immunostaining (Figure 5). The 3X FUTP group showed a significantly (*p* < 0.05) higher blastocyst development rate than did the FUTP group (77.2% ± 2.3% vs. 63.1% ± 2.9%). The 3X FUTP group showed a significantly (*p* < 0.05) higher total cell number in the blastocyst than did the control and FUTP groups (71.3% ± 3.9% vs. 39.2% ± 2.5% vs. 29.8% ± 2.1%, respectively). The FUTP group showed a significantly (*p* < 0.05) lower total number of blastocyst cells than did the control group. The FUTP and 3X FUTP groups showed a significantly (*p* < 0.05) lower ICM ratio than did the control group (8.2%–10.1% vs. 14.7%) and a significantly higher TE cell ratio (89.7%–92.5% vs. 85.3%).

### 3.6 Expression of pluripotency and TE cell markers genes in aggregated embryo-derived blastocysts

**FIGURE 6:**
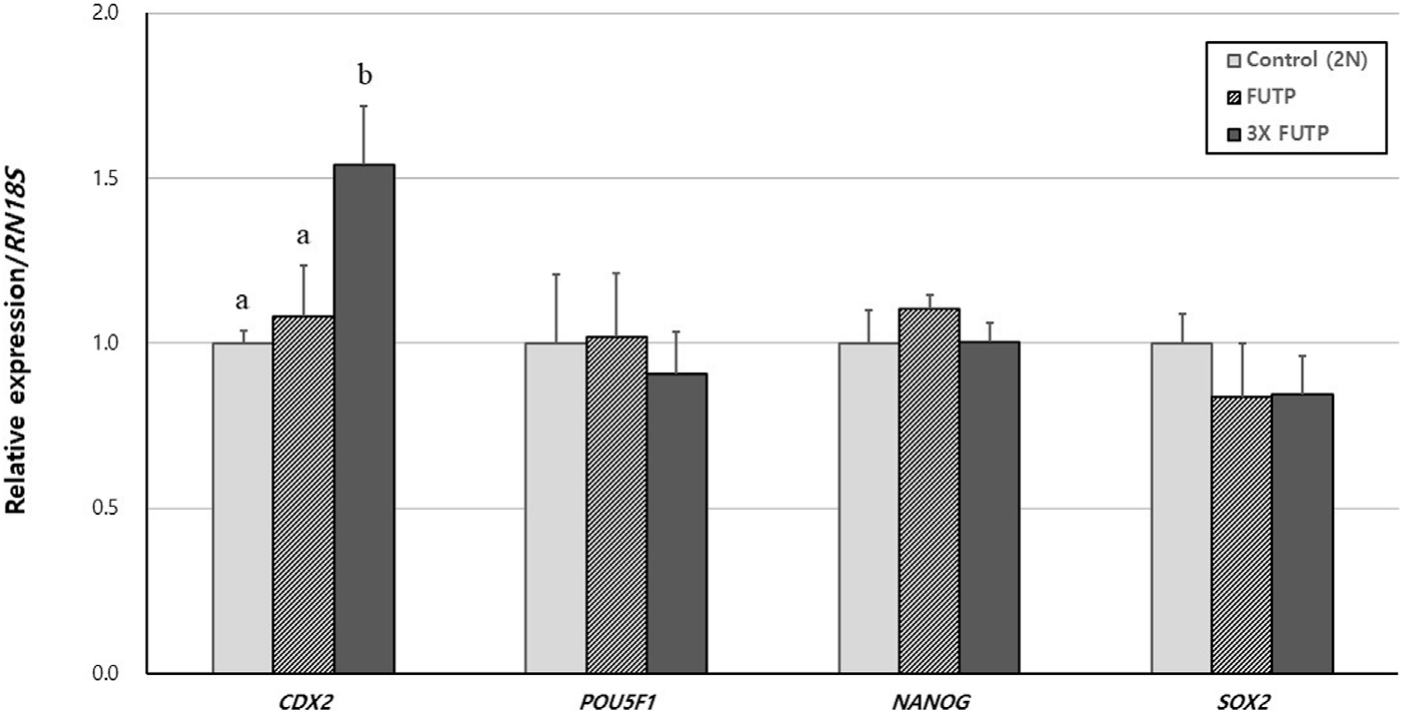
Relative expression of porcine pluripotency-related genes and trophectoderm markers in aggregated tetraploid blastocysts. The mRNA levels of the genes were analyzed using quantitative real-time polymerase chain reaction, with three replicates per sample. The expression of each gene was normalized against *RN18S* mRNA. Data are the mean ± SEM. ^a, b^ Values in the same column with different superscript letters are significantly different (*p* < 0.05).

Using RT-PCR, we investigated the relative gene expression of pluripotency markers *POU5F1*, *NANOG*, *SOX2,* and TE cell marker *CDX2* in blastocysts (Figure 6). Expression of the *CDX2* gene was significantly (*p* < 0.05) higher in the 3X FUTP group than in the control and FUTP groups. There was no significant difference in the relative expression of the pluripotency markers *POU5F1*, *NANOG*, and *SOX2* genes in all groups.

### 3.7 Distribution of YAP1 protein in aggregated embryo-derived blastocysts

**FIGURE 7:**
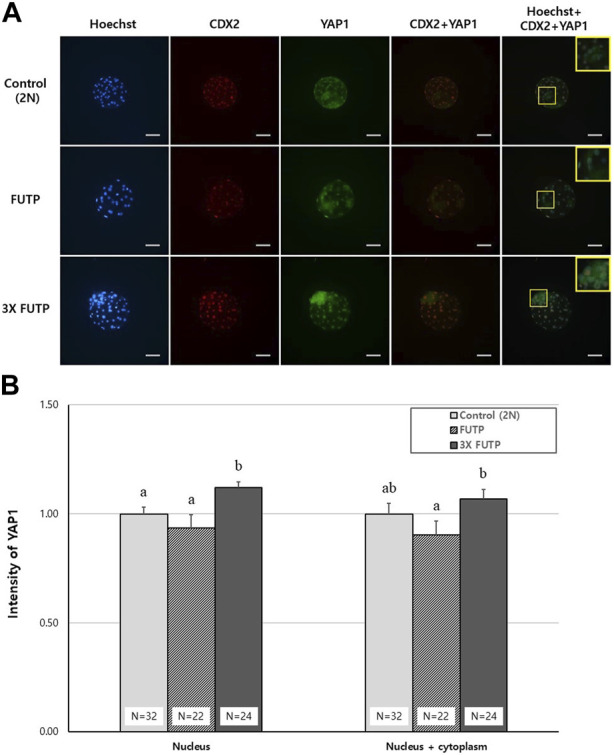
YAP1 protein expression intensity in the whole blastocyst and nucleus through immunostaining. **(A)** Representative images of blastocysts from each treatment group immunostained with Hoechst (blue), CDX2 (red), and YAP1 (green). The yellow box is a two-fold magnified image of the ICM region of the blastocyst. Scale bar = 50 μm. **(B)** The blastocysts from each treatment group were examined for YAP1 expression in the entire cytoplasm, including the nucleus or nucleus, through YAP1 immunostaining. N = the number of blastocysts analyzed. Data are the mean ± SEM. ^a, b^ Values in the same column with different superscript letters are significantly different (*p* < 0.05).

The blastocysts derived from each treatment group were examined for YAP1 protein expression intensity in the whole blastocyst and nucleus through immunostaining (Figure 7A). The 3X FUTP group showed significantly (*p* < 0.05) higher YAP1 expression in the nucleus than did the control and FUTP groups. In the cytoplasm of the entire blastocyst, including the nucleus, the 3X FUTP group showed significantly (*p* < 0.05) higher YAP1 expression than did the FUTP group, but there was no significant difference in the control group (Figure 7B).

## 4 Discussion

Tetraploid complementation is a method that ideally proves the differentiation potential to differentiate into all tissue cells except for placental tissue. In other words, when pluripotent stem cells and placenta-forming cells are combined and implanted, an individual composed of 100% pluripotent stem cell-derived cells can be created. To make this practically possible, the TE that forms the placenta and pluripotent stem cells should be combined and implanted. To facilitate this through experimentation, a more stable supply of tetraploid blastocysts must be established. However, there is limited evidence to measure gene products in tetraploid mammalian cells (Eakin and Behringer, 2003).

In this study, PA embryos were used to produce tetraploid embryos. Although PA embryos cannot maintain normal pregnancy, it is a good material for observing developmental competence and characteristics in the preimplantation embryo stage. IVF embryos can be used for this study, but in the case of pigs, the fertility rate varies greatly depending on the quality of semen. Furthermore, a high polyspermy rate of porcine IVF embryos increases karyotypic abnormalities (McCauley et al., 2003; Nguyen et al., 2020), which can be an obstacle to analyzing the efficiency of tetraploid production. The tetraploid production technology established in this study can use pig embryos derived from IVF or somatic cell nuclear transfer in the future.

In the first experiment, there was no significant difference in cleavage rate in all treatment groups after 24 h of electrical stimulation for PA. However, blastocysts derived from the CITP group showed a significantly lower blastocyst development rate than did the blastocysts derived from the control group (diploid). In addition, the fact that the CITP group shows differences in fusion rate and blastocyst quality depending on the type of donor cells injected may be a disadvantage in the production of tetraploid embryos.

In the second experiment, we investigated the nuclear ploidy of blastocysts from each treatment group. In the control group, 84% of the blastocysts were diploid. The tetraploid ratio of blastocysts derived from FUTP, CITP, and CBTP was also investigated at 74.6%–86%, showing high tetraploid formation in all methods. Several previous studies have also shown that these methods have a high tetraploid formation rate (Sembon et al., 2011; Kong and Liu, 2019).

A study by Lin et al. reported that there was no significant difference in the frequency of apoptotic cells in tetraploid blastocysts produced through CBTP and FUTP methods (Lin et al., 2019). However, our results showed that blastocysts derived from the CITP-treated group and CBTP-treated group showed a significantly higher percentage of apoptotic cells than the FUTP-treated group and the control group. For the oocytes used in the other treatment groups except for the CBTP treatment group; only MII oocytes are selected for PA after IVM. Hence, oocytes that do not spontaneously mature to MII are filtered out. However, CBTP-derived oocytes in which extrusion of the PB1 is inhibited by CB treatment did not filter out oocytes that did not naturally mature. These oocytes may have been responsible for the high rate of apoptosis when they reach the blastocyst. The low developmental capacity of somatic cell nuclear transfer has been primarily attributed to incomplete reprogramming of the donor (Zhang et al., 2020; Glanzner et al., 2022). Donor cells injected for the production of CITP-derived tetraploid embryos also have processing similar to somatic cell nuclear transfer. Factors such as the synchrony of the cell cycle stages of the donor cells, various ages, and tissue origin may have caused low reprogramming and increased the rate of cell death. Based on the results of experiments 1, 2, and 3, we selected the FUTP method as the most suitable method for producing porcine tetraploid embryos.

The size of a tetraploid blastocyst remains approximately the same as that of a diploid blastocyst at the same stage (Edwards, 1958; Eakin and Behringer, 2003). In the results of the fourth experiment, the size of the tetraploid blastocyst remained almost the same as that of the diploid blastocyst at the same stage. However, it was confirmed that the average cell nucleus diameter of tetraploid-derived blastocysts was significantly larger than that of diploids. This result can be attributed to two reasons. The size of the nucleus gradually decreases during preimplantation development in mice (Tsichlaki and FitzHarris, 2016), which means that tetraploid blastocysts can have large nuclei because the cell cycle is slower than that of diploids. Another reason is that tetraploid blastocysts are similar in size to diploid blastocysts but contain fewer cells. This means that the cells of a tetraploid blastocyst constitute a larger area of blastocyst surface area per cell. Based on the morphological values of the blastocysts measured in this experiment, the relative area ratio of 1 cell constituting the blastocyst was calculated. As a result, it was determined that the cells of the tetraploid blastocyst cover areas 1.34 to 1.43 times more than the cells of the diploid blastocyst. Although no similar references can be found for pigs, it is evident from other literature that used mouse embryos, which reported that the size of the cell nucleus in the early preimplantation embryo is affected by the size of the cytoplasm (Tsichlaki and FitzHarris, 2016). Therefore, it can be assumed that the nucleus of a tetraploid with a relatively large cytoplasm is larger than that of a diploid blastocyst.

There have been reports in pigs that aggregation of diploid embryos improves blastocyst quality (Terashita et al., 2011; Buemo et al., 2016), but this has not been reported for cases of tetraploid embryos. In the fifth experiment, we demonstrated that the aggregation of porcine tetraploid embryos increases the rate of development into blastocysts and the number of cells in blastocysts. The tetraploid blastocyst showed a significantly higher TE cell ratio than did the diploid blastocyst (control), and the ratio of ICM and TE did not change depending on blastomere aggregation. As the embryo develops from a morula to a blastocyst, it divides into ICM and TE. In the sixth experiment, *CDX2* mRNA expression was significantly higher in the 3X FUTP group with the highest number of TE cells than in the other groups. This result demonstrates that there is a close relationship between high *CDX2* expression and the number of TE cells in aggregated tetraploid embryos, probably because most of the cells in the tetraploid blastocyst must consist of outer cells to construct a similar size to the diploid blastocyst, with a relatively small number of cells compared to the diploid. Cells inside the morula differentiate into ICM expressing *OCT4* (*POU5F1*) and *NANOG*, and cells outside the morula differentiate into trophoblast expressing CDX2. This mechanism originates from the inside-outside hypothesis of Tarkowski and Wróblewska (1967) (Tarkowski and Wróblewska, 1967).

We further investigated whether the high percentage of TE cells in aggregated tetraploid blastocysts was due to outer cell origin. During the development of the mammalian embryo, differentiation of the ICM and TE during the transition from morula to blastocyst is regulated by the Hippo signaling pathway (Pedersen et al., 1986; Karasek et al., 2020). However, the function of the Hippo signaling pathway in porcine embryogenesis remains relatively unexplored.

Hippo pathway is known to play important roles in the ICM/TE decision in murine (Nishioka et al., 2009; Hirate et al., 2013). The downstream effector YAP1 is the core component of the Hippo pathway. At the morula stage, the Hippo pathway is quiescent in the outer cells, and YAP1 is not phosphorylated by Lats1/2 kinases. Thus, YAP1 can localize to the nucleus and combine with Tead4 to induce the expression of TE-related genes such as *CDX2* (Yagi et al., 2007; Nishioka et al., 2008; Nishioka et al., 2009; Home et al., 2012; Rayon et al., 2014). In our seventh experiment, the expression of the YAP1 protein was more clearly observed in the nuclei of TE cells, and most SOX2-positive nuclei were YAP1-negative. This trend is consistent with the results of Emura et al. that in porcine blastocyst stage embryos, nuclear YAP1 signals were observed at least in TE cells (Emura et al., 2020). Aggregated tetraploid blastocysts with an increased proportion of TE cells can display higher efficiency in implantation and placenta formation after tetraploid complementation using pigs. However, this study has limitations in that the results were obtained using PA embryos that cannot develop into complete organisms. Another limitation is that the efficiency of embryos produced through condensed tetraploid blastocyst complementation was not verified *in vivo*. Therefore, further studies are needed to determine whether tetraploid aggregated blastocysts derived from *in vivo* embryos or IVF embryos can sustain intact pregnancies when used in tetraploid complementation.

This study demonstrated that the FUTP method was the most efficient method for producing tetraploid blastocysts in pigs with a low apoptosis rate. Tetraploid embryos had fewer cells in the blastocyst than did diploid embryos, but this disadvantage can be overcome through tetraploid embryo aggregation. In addition, the aggregated FUTP-treated group maintained a high ratio of TE based on the increased expression of the *CDX2* gene and high YAP1 intensity in the nucleus. The established method could improve the quality of donor blastocysts used in tetraploid complementation and provide insight into understanding the evolution and regulation of pluripotency across mammalian species.

## Data Availability

The original contributions presented in the study are included in the article/Supplementary Material, further inquiries can be directed to the corresponding authors.

## Abbreviations

AI/TI, Anaphase I/telophase I; CB, Cytochalasin B; CoCs, Cumulus oocyte complexes; ES, Embryonic stem cells; GV, Germinal vesicles; iPS, Induced pluripotent stem cells; IVC, *In vitro* culture medium; MI, Metaphase I; MII, Metaphase II; PA, parthenogenetic activation; PHA-L, Phytohemagglutinin-L; PZM-3, Porcine zygote medium; qRT-PCR, Quantitative real-time polymerase chain reaction; TE, Trophectoderm; TS, Tyrode′s Solution.
