# Supplementary material for: Tetraploid embryo aggregation produces high-quality blastocysts with an increased trophectoderm in pigs
Source: Front Cell Dev Biol. 2023 Nov 16;11:1239448. doi: 10.3389/fcell.2023.1239448 (PMC10687364; doi:10.3389/fcell.2023.1239448)
Supplement: Supplementary file 1 [file Table1.docx]

Supplementary Material

Tetraploid embryo aggregation produces high-quality blastocysts with an increased trophectoderm in pigs

**Joohyeong Lee^1^, Lian Cai^2, 3, 4^, Mirae Kim^2, 3^, Hyerin Choi^2, 3^, Dongjin Oh^2, 3^, Ali Jawad^2, 3^, Eunsong Lee^5^, Sang-Hwan Hyun^2, 3, 4, *^**

^1^Department of Companion Animal Industry, College of Healthcare & Biotechnology, Semyung University, Jecheon 27136, Republic of Korea

^2^Veterinary Medical Center and College of Veterinary Medicine, Laboratory of Veterinary Embryology and Biotechnology (VETEMBIO), Chungbuk National University, Cheongju 28644, Republic of Korea

^3^Institute of Stem Cell & Regenerative Medicine (ISCRM), Chungbuk National University, Cheongju 28644, Republic of Korea

^4^Graduate School of Veterinary Biosecurity and Protection, Chungbuk National University, Cheongju 28644, Republic of Korea

^5^College of Veterinary Medicine, Kangwon National University, Chuncheon 24341, Republic of Korea

***Correspondence:**Sang‐Hwan Hyun

shhyun@cbu.ac.kr

# Supplementary Tables

Supplementary Table 1. Primers used for gene expression analyses

Supplementary Table 2. Antibody lists for immunofluorescence analysis

Supplementary Table 3. Meiotic maturation of porcine oocytes treated with Cytochalasin B during the last 22–44 h of in vitro maturation

Supplementary Table 4. Developmental competence of tetraploid blastocysts produced by injection of different types of donor cells

| **Supplementary Table 1.** Primers used for gene expression analyses | | | | | |
| --- | --- | --- | --- | --- | --- |
| Gene | Primer sequences (5′→3′) | | Product size (bp) | Tm (°C) | GenBank accession number |
| *RN18S* | F: | CGCGGTTCTATTTTGTTGGT | 219 | 56 | NR_046261.1 |
|  | R: | AGTCGGCATCGTTTATGGTC |  | 58 |  |
| *CDX2* | F: | CTGTTTGGGTTGTTGGTCTG | 95 | 59 | NM_001278769.1 |
|  | R: | CCCACTCCCTTCACCATATC |  | 60 |  |
| *POU5F1* | F: | GCGGACAAGTATCGAGAACC | 200 | 58 | XM_021097869.1 |
|  | R: | CCTCAAAATCCTCTCGTTGC |  | 57 |  |
| *NANOG* | F: | TAAAACCACTGCCCACATCT | 131 | 57 | XM_021092390.1 |
|  | R: | CTGCCTCTGAAATCTGTCGT |  | 58 |  |
| *SOX2* | F: | CCGTGGTTACCTCTTCTTCC | 186 | 58 | NM_001123197.1 |
|  | R: | AGAGAGGCAGTGTACCGTTG |  | 59 |  |
| Tm: temperature | | | | | |

| **Supplementary Table 2.** Antibody list for immunofluorescence analysis | | | |
| --- | --- | --- | --- |
| Primary antibody | Host species | Dilution | Catalog number |
| SOX2 | Rabbit | 1:200 | AB5603 |
| CDX2 | Rabbit | 1:200 | 12306S |
| YAP1 | Mouse | 1:100 | MAB8094 |

| **Supplementary Table 3.** Meiotic maturation of porcine oocytes treated with Cytochalasin B during the last 22–44 h of *in vitro* maturation | | | | | |
| --- | --- | --- | --- | --- | --- |
| Treatment | No. of matured oocytes* | Nuclear status (%) | | | |
|  |  | Germinal Vesicle | Metaphase I | Anaphase I & Telophase I | Metaphase II |
| Control | 216 | 1.2 ± 0.8 | 8.0 ± 2.2 ^a^ | 0.4 ± 0.4 | 90.4 ± 2.5 ^a^ |
| Cytochalasin B ^A^ | 100 | 0.0 ± 0.0 | 96.7 ± 1.2 ^b^ | 0.0 ± 0.0 | 3.3 ± 1.2 ^b^ |
| *Four replicates.  ^A^ Oocytes matured with 5 µg/mL Cytochalasin B during last 22 hours of *in vitro* maturation.  ^a, b^ Values in the same column with different superscript letters are significantly different (*p* < 0.01). | | | | | |

| **Supplementary Table 4.** Developmental competence of tetraploid blastocysts produced by injection of different types of donor cells | | | | | |
| --- | --- | --- | --- | --- | --- |
| Type of donor cell | No. of embryos cultured* | No. (%) of fused with donor-oocytes | No. (%) of 2-cell | No. (%) of embryos developed to blastocyst/2-cell | No. of cells in blastocyst |
| Fibroblast | 77 | 56 (73.2 ± 3.8) ^a^ | 38 (67.8 ± 2.3) | 12 (38.3 ± 2.7) | 29.2 ± 4.7 ^a^ |
| Cumulus | 127 | 64 (53.1 ± 8.0) ^b^ | 46 (72.2 ± 6.9) | 16 (38.3 ± 0.0) | 58.5 ± 7.3 ^b^ |
| *Three replicates.  ^a, b^ Values in the same column with different superscript letters are significantly different (*p* < 0.05). | | | | | |

**Supplementary Figure**

Supplementary Figure 1. Formulas for diameter, radius, and surface area of the blastocyst, and surface area per cell.


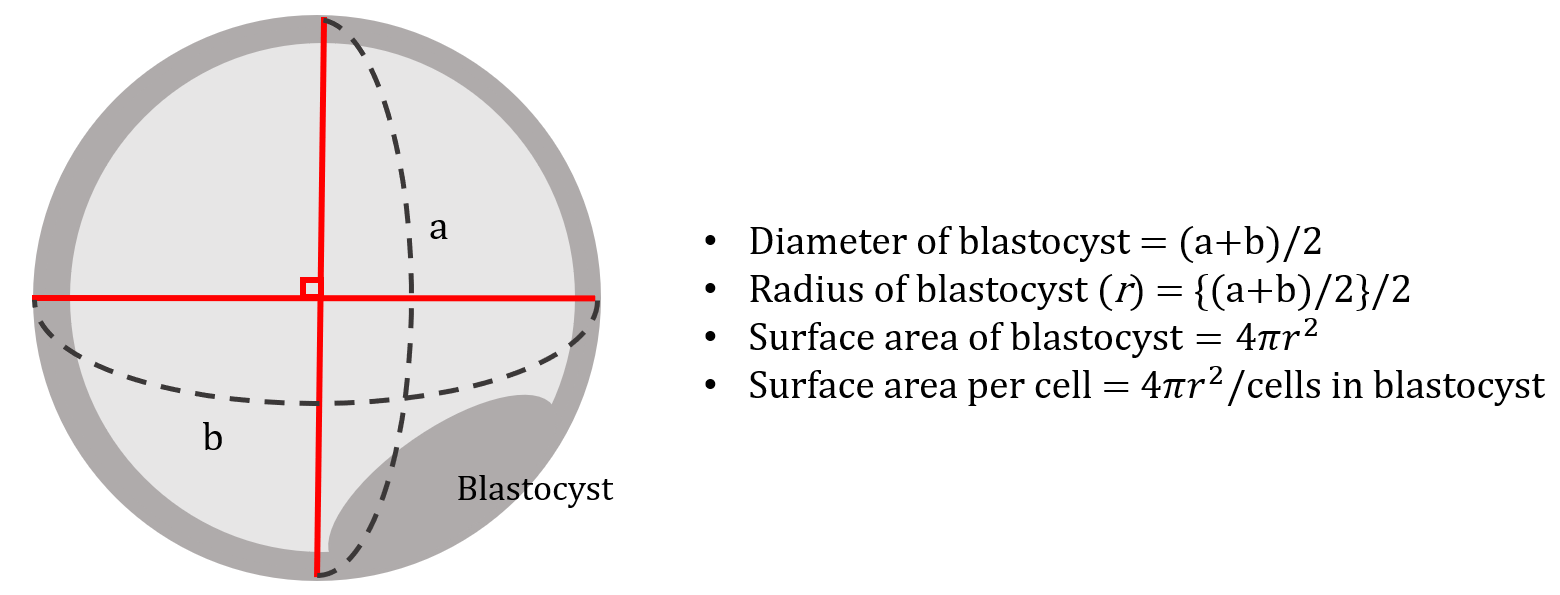


Supplementary Figure 1. Formulas for diameter, radius, and surface area of the blastocyst, and surface area per cell.
